# Two Gene Set Variation Index as Biomarker of Bacterial and Fungal Sepsis

**DOI:** 10.1155/2020/8182358

**Published:** 2020-06-03

**Authors:** Xiaowen Zheng, Yifeng Luo, Qian Li, Jihua Feng, Chunling Zhao, Junyu Lu, Jiefeng Luo, Jianfeng Zhang

**Affiliations:** Department of Emergency Medicine, The Second Affiliated Hospital of Guangxi Medical University, Nanning 530007, China

## Abstract

**Background:**

The incidence of sepsis has been increasing in recent years. The molecular mechanism of different pathogenic sepsis remains elusive, and biomarkers of sepsis against different pathogens are still lacking.

**Methods:**

The microarray data of bacterial sepsis, fungal sepsis, and mock-treated samples were applied to perform differentially expressed gene (DEG) analysis to identify a bacterial sepsis-specific gene set and a fungal sepsis-specific gene set. Functional enrichment analysis was used to explore the body's response to bacterial sepsis and fungal sepsis. Gene set variation analysis (GSVA) was used to score individual samples against the two pathogen-specific gene sets, and each sample gets a GSVA index. Receiver operating characteristic (ROC) curve analysis was performed to evaluate the diagnostic value of sepsis. An independent data set was used to validate the bacterial sepsis-specific GSVA index.

**Results:**

The genes differentially expressed only in bacterial sepsis and the genes differentially expressed only in fungal sepsis were significantly involved in different biological processes (BPs) and pathways. This indicated that the body's responses to fungal sepsis and bacterial sepsis are varied. Twenty-two genes were identified as bacterial sepsis-specific genes and upregulated in bacterial sepsis, and 23 genes were identified as fungal sepsis-specific genes and upregulated in fungal sepsis. ROC curve analysis showed that both of the two pathogen sepsis-specific GSVA indexes may be a reliable biomarker for corresponding pathogen-induced sepsis (AUC = 1.000), while the mRNA of CALCA (also known as PCT) have a poor diagnostic value with AUC = 0.512 in bacterial sepsis and AUC = 0.705 in fungi sepsis. In addition, the AUC of the bacterial sepsis-specific GSVA index in the independent data set was 0.762.

**Conclusion:**

We proposed a bacterial sepsis-specific gene set and a fungal sepsis-specific gene set; the bacterial sepsis GSVA index may be a reliable biomarker for bacterial sepsis.

## 1. Introduction

Sepsis is a life-threatening disease characterized by systemic inflammation caused by infection [1–3]. It usually occurs after infection with virus, fungi, and bacteria [4]. At present, sepsis incidence is estimated at 270 cases per 100,000 persons/year followed by an approximately 26% mortality rate, potentially 5.3 million deaths annually in the world [5, 6]. In recent years, great progress has been made in searching for biomarkers of sepsis, including CALCA (also known as PCT) [7], C-reactive protein [8, 9], and interleukin-6 [10] which have been found to be biomarkers of sepsis. However, their clinical application is limited [11] due to these biomarkers that are nonpathogen-specific sepsis. Since the most common two types of pathogens, bacterial and fungal, require fundamentally different therapies, the classification is crucial in the management of sepsis. Sepsis is characterized as a host reaction to infection involving not only the activation of pro- and anti-inflammatory responses but also modifications in nonimmunological pathways (cardiovascular, autonomic, neurological, hormonal, metabolic, and clotting) [12]. We hypothesized that the reactions of the host to bacterial and fungal sepsis may be different, and the different reactions may be reflected in the whole blood gene expression patterns. To explore our hypothesis and provide potential pathogen-specific biomarkers to help distinguish bacterial and fungal sepsis. In the present study, the gene expression profiles of bacterial and fungal sepsis were used for analysis. Compared to mock-treated blood, the differentially expressed genes (DEGs) in bacterial and fungal sepsis were, respectively, screened. Functional enrichment analysis was performed to explore the body's reactions to sepsis induced by different pathogens. Moreover, we identified and validated a gene set as biomarkers of bacterial sepsis.

## 2. Materials and Methods

### 2.1. Data Collection and Processing

**Figure 1:**
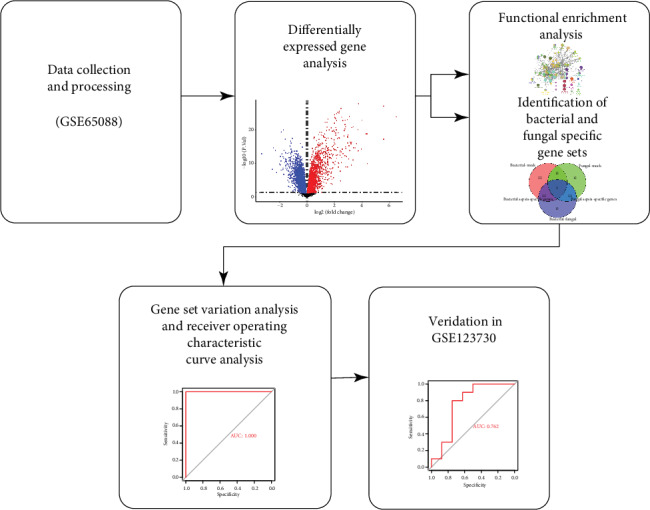
The workflow of the present study.

In the present study, the whole blood gene expression profiles of GSE65088 [13] in the website of GEO (https://http://www.ncbi.nlm.nih.gov/) were downloaded, including 20 bacterial sepsis (10 Escherichia coli and 10 Staphylococcus aureus), 16 fungal sepsis (6 Aspergillus fumigatus and 10 Candida albicans), and 21 mock-treated whole blood samples. The whole blood gene expression profiles of GSE123730 [14] were downloaded to validate the gene set, including 10 bacterial sepsis and 35 nonsepsis. The normalizeBetweenArrays function in limma package [15] was used to normalize the gene expression profile. This gene expression profile of GSE65088 was based on GPL10558 while GSE123730 was based on GPL19803. If a gene corresponds to multiple probes, the average expression value of these probes is the expression value of the gene. The workflow of the present study is shown in Figure 1.

### 2.2. DEGs in Bacterial Sepsis and Fungal Sepsis

Compared to the mock-treated samples, the DEGs in bacterial sepsis and fungal sepsis samples were, respectively, screened using the limma package in R. The fold changes (FCs) in the expression of individual genes were calculated, and genes with ∣logFC | >2 and *P* < 0.01 adjusted by the false discovery rate (FDR) were considered significant.

### 2.3. Functional Enrichment Analysis

To explore the difference of human response to bacterial sepsis and fungi sepsis. The ClueGO plugin [16] of Cytoscape [17] software was applied to create a functionally organized biological process (BP) term network of DEGs only in bacterial sepsis, fungal sepsis, and common DEGs. Kyoto Encyclopedia of Genes and Genomes (KEGG) pathway enrichment analyses of DEGs only in bacterial sepsis, fungal sepsis, and common DEGs were performed, respectively, using the clusterProfiler package [18].

### 2.4. Identification of Pathogen-Specific Gene Set in Sepsis

The DEGs in bacterial sepsis compared with those in fungal sepsis were also screened using the same method and the same threshold. Subsequently, the VennDiagram package [19] in R was performed to identify pathogen-specific DEGs of bacterial sepsis and fungal sepsis in DEG bacterial sepsis vs. mock-treated, DEG fungal sepsis vs. mock-treated, and DEG bacterial sepsis vs. mock treated. We generated two gene sets for bacterial sepsis and fungal sepsis, respectively. Gene set variation analysis (GSVA) [20] was used to score individual samples against the gene set, and each sample set gets a bacterial sepsis-specific GSVA index and fungal bacterial sepsis-specific GSVA index. The GSVA package in R was used in the GSVA.

### 2.5. Receiver Operating Characteristic (ROC) Curve Analysis

ROC curve analysis was performed to access the diagnostic value for sepsis of the two pathogen-specific sepsis GSVA indexes. ROC curve analysis was performed using pROC package [21] in R. In addition, although protein of CACLA has been identified and used in clinic as a biomarker of sepsis [7, 22], the diagnostic value of mRNA of CACLA was also evaluated in the present study.

### 2.6. Validation of Bacterial Sepsis-Specific GSVA Index in an Independent Data Set

As it was in the GSE65088, the bacterial sepsis-specific GSVA index was calculated for the samples in GSE123730. ROC curve analysis was performed to evaluate the value of bacterial sepsis-specific GSVA index and the mRNA of CALCA for diagnosing bacterial sepsis.

## 3. Results and Discussion

### 3.1. The Gene Expression Patterns of Whole Blood Were Varied in Bacterial Sepsis and Fungal Sepsis

**Figure 2:**
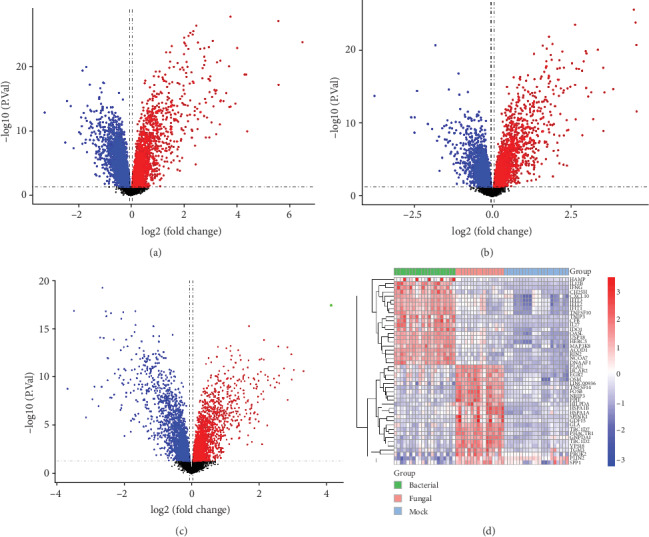
Differentially expressed gene (DEG) analysis. (a) Volcano plot of the DEGs in bacterial sepsis. (b) Volcano plot of the DEGs in fungal sepsis. (c) Volcano plot of the DEGs in bacterial versus fungal sepsis. Red indicates upregulated and blue indicates downregulated. (d) The expression patterns of DEGs that can basically distinguish bacterial sepsis and fungal sepsis.

Compared to mock-treated samples, a total of 95 DEGs were found in bacterial sepsis, 90 of which were upregulated and 5 of which were downregulated (Figure 2(a)). In fungal sepsis, a total of 56 DEGs were found, 50 of which were upregulated, and 6 of which were downregulated (Figure 2(b)). In bacterial sepsis versus fungal sepsis, 61 DEGs were found totally, 28 of which were upregulated and 33 of which were downregulated (Figure 2(c)). The expression heat map showed that the gene expression patterns may distinguish bacterial sepsis and fungal sepsis (Figure 2(d)) from mock samples.

### 3.2. Enrichment Analysis of DEGs

**Figure 3:**
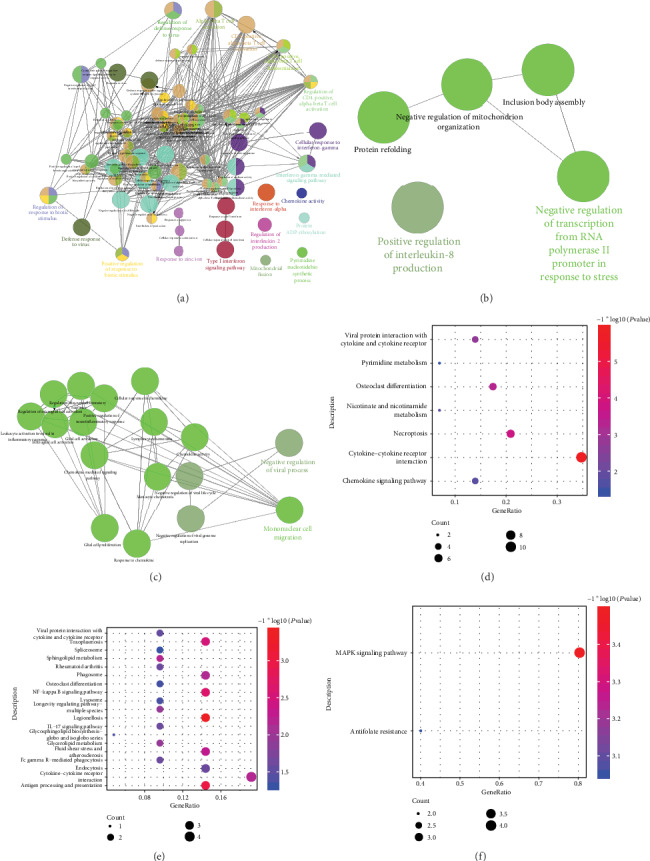
Results of functional enrichment. (a) The biological processes in which the DEGs of bacterial sepsis were involved in. (b) The biological processes in which the DEGs of fungal sepsis were involved in. (c) The biological processes in which the common DEGs were involved in. (d) The KEGG pathway of the DEGs of bacterial sepsis involved in. (e) The KEGG pathway of the DEGs of fungal sepsis involved in. (f) The KEGG pathway of the common DEGs involved in.

Functional enrichment analysis was used to explore the difference of the host reaction to bacterial sepsis and fungal sepsis. The results of ClueGO analysis revealed that the genes only differentially expressed in bacterial sepsis were significantly involved in defense response to virus, regulation of defense response to virus, and positive regulation of response to biotic stimulus and other biological processes (Figure 3(a)), while the genes only differentially expressed in fungal sepsis were significantly enriched in the biological processes such as negative regulation of transcription from RNA polymerase II promoter in response to stress and positive regulation of interleukin-8 production and other biological processes (Figure 3(b)). The common DEGs in bacterial sepsis and fungal sepsis were significantly enriched in BP mononuclear cell migration and negative regulation of viral process (Figure 3(c)).

The genes only differentially expressed in bacterial sepsis are significantly involved in viral protein interaction with cytokine and cytokine receptor, chemokine signaling pathway, and necroptosis pathways (Figure 3(d)). The genes only differentially expressed in fungal sepsis are significantly involved in antigen processing and presentation and lysosome and NF-kappa B signaling pathway (Figure 3(e)). The genes both differentially expressed in bacterial and fungal sepsis were significantly involved in MAPK signaling pathway and antifolate resistance (Figure 3(f)).

### 3.3. Bacterial Sepsis- and Fungal Sepsis-Specific Gene Sets

**Figure 4:**
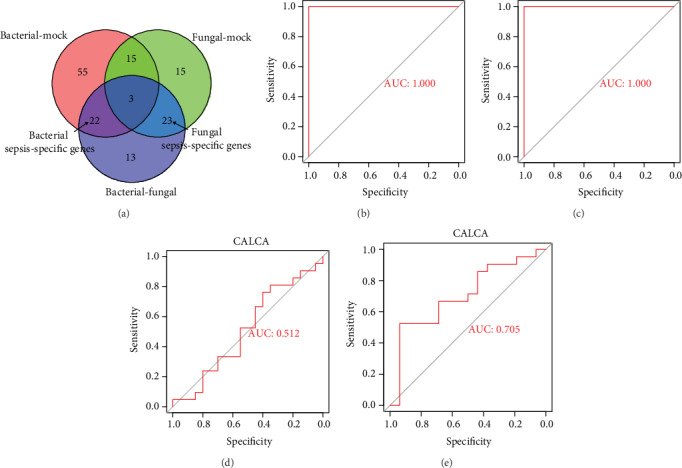
Pathogen sepsis-specific gene set and ROC curve analysis. (a) VennDiagram was used to identify bacterial sepsis-specific genes and fungal sepsis-specific genes. (b) The ROC curve result of bacterial sepsis-specific GSVA index for bacterial sepsis in GSE65088. (c) The ROC curve result of fungal sepsis GSVA index for fungal sepsis in GSE65088. (d) The result of ROC of CALCA mRNA for bacterial sepsis in GSE65088. (e) The result of ROC of CALCA mRNA for fungal sepsis in GSE65088.

A gene set included 22 bacterial sepsis-specific DEGs, and a gene set included 23 fungal sepsis-specific DEGs were generated, respectively (Figure 4(a)). Compared to the mock-treated samples, all the 22 bacterial sepsis-specific genes (HAMP, IL12B, IFNG, CH25H, CXCL10, IFIT3, IFIT2, IFIT1, TNFSF10, TNIP3, CFB, IL27, IDO1, OASL, USP18, HERC5, MAP3K8, ACOD1, RIN2, NCOA7, DNAAF1, and PLAU) were upregulated in bacterial sepsis, and all the 23 fungal sepsis-specific genes (HCAR2, EFR2, OSM, LINC00936, TNFSF14, FOSB, NRIP3, PPIF, HILPDA, HSPA1B, HSPA1A, SPINK1, GDF15, GLA, TBC1D7, PHACTR1, GNPDA1, TBC1D2, VPS18, TGM3, PROK2, PLIN2, and SPP1) were also upregulated in fungal sepsis.

Subsequently, all samples got a bacterial sepsis-specific GSVA index and a fungal sepsis-specific sepsis GSVA index. ROC curve analysis showed that the two pathogen-specific sepsis GSVA indexes may be powerful biomarkers for bacterial sepsis (Figure 4(b)) and fungal sepsis (Figure 4(c)) with both AUC = 1.000, while the mRNA of CALCA may not be a good biomarker of sepsis in bacterial sepsis with an AUC =0.512 (Figure 4(d)) and fungal sepsis with an AUC = 0.705 (Figure 4(e)).

### 3.4. Validation of the Bacterial Sepsis-Specific GSVA Index

**Figure 5:**
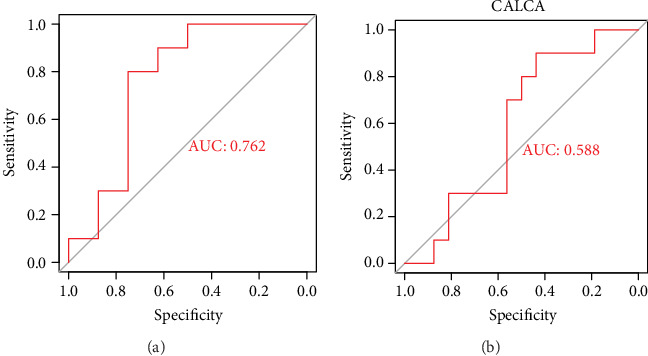
ROC curve of the bacterial sepsis-specific GSVA index and CALCA in GSE123730. (a) The ROC curve result of bacterial sepsis-specific GSVA index in GSE123730. (b) ROC curve of CALCA in GSE123730.

In GSE123730, the ROC curve analysis showed that the bacterial sepsis-specific GSVA index may also be a reliable biomarker with AUC = 0.762 (Figure 5(a)), while the mRNA of CALCA remained to have a poor diagnostic value for bacterial sepsis with AUC = 0.588 (Figure 5(b)). This result was consistent with that in GSE65088.

Sepsis accounts for an estimated 30 million cases and 6 million deaths globally each year. [23] However, the mechanism of sepsis was not fully understood. Though there are some biomarkers that have been found, a reliable biomarker of pathogen-specific sepsis remains an unmet medical need. It is often difficult to distinguish between bacterial and fungal sepsis early in suspected sepsis, and the difficulty in distinguishing between bacterial and nonbacterial aetiologies is also a cause of the misuse of antibiotics [24] and contributes to the emergence of antibiotic resistance [25]. In present study, we found the gene expression patterns of the whole blood were varied in bacterial sepsis and fungal sepsis; this opens up the possibility of using whole blood gene expression profiles to distinguish between types of sepsis caused by different pathogens.

Functional enrichment analysis revealed that the body's responses to sepsis induced by different pathogens were also different. The BPs of negative regulation and mononuclear cell migration, the KEGG pathways of MAPK signaling pathway, and antifolate resistance were enriched in bacterial sepsis and fungal sepsis; these body responses were common in bacterial and fungal sepsis. This may provide potential therapeutic targets for sepsis when the pathogens are not confirmed. The BPs enriched in bacterial-sepsis mitochondrial fission, protein ADP-ribosylation, and chemokine activity may be the specific reactions of the body to bacterial sepsis, while the BPs enriched in fungal sepsis of positive regulation of interleukin-8 production and negative regulation of transcription from RNA polymerase II promoter in response to stress were the specific response of the body to fungal sepsis. The pathways of viral protein interaction with cytokine and cytokine receptor, chemokine signaling pathway [26], and necroptosis [27, 28] were significantly enriched in bacterial sepsis, while the pathways of the antigen processing and presentation [29], lysosome, and NF-kappa B signaling pathway were significantly enriched in fungal sepsis, and these three pathways may be the body's immune response to fungal sepsis [30–32]. We have preliminarily revealed the response of the human immune system to sepsis caused by different pathogens.

Furthermore, a bacterial sepsis-specific gene set and a fungal sepsis-specific gene set were generated in our present study. In the bacterial sepsis-specific gene set, unsurprisingly, some of them were reported to be associated with bacterial sepsis, such as IFNG, CXCL10 [33], HERC5 [34], IFIT2 [35], TNFSF10 [36], CFB [37], IL27 [38], IDO1 [39], MAP3K8 [40], and PLAU [41]. And OSM [42], HSPA1A [43], GDF15 [44], GLA [45], and PROK2 [46] in the fungal sepsis-specific gene set were reported to be associated with fungal sepsis. Our present study indicated that HAMP, IL12B, CH25H, IFIT1, TNIP3, ACOD1, RIN2, NCOA7, and DNAAF1 may also be associated with bacterial sepsis, while HCAR2, EFR2, LINC00936, TNFSF14, FOSB, NRIP3, PPIF, HILPDA, HSPA1B, SPINK1, TBC1D7, PHACTR1, GNPDA1, TBC1D2, VPS18, TGM3, PLIN2, and SPP1 may also be associated with fungal sepsis. These may be potential candidate hub molecules for sepsis.

Moreover, a bacterial sepsis-specific GSVA index and a fungal sepsis-specific GSVA were calculated in the present study. To our best knowledge, it was the first time to use the method of single sample gene set enrichment analysis to investigate the underlying mechanism of sepsis. We found that the two sepsis GSVA score systems may be reliable biomarkers for sepsis, especially, the bacterial sepsis GSVA index been validated in another independent data set. In addition, though PCT emerged as the leading biomarker to indicate the presence of systemic infection [22], the diagnostic value of mRNA of CALCA was poor. This indicated that the transcriptional regulation of sepsis is complex.

Although our study may provide new insights for sepsis, there are several notable limitations. The cost of sequencing is still high which may limit clinical application now, but the cost of sequencing is declining. Due to the fact that fungal sepsis is relatively rare in clinical practice, it will take a long time to collect data in a single center. We also failed to find a suitable validation set in the GEO database; thus, the fungal sepsis GSVA were not validated in the present study.

## 4. Conclusions

We proposed a bacterial sepsis-specific gene set and a fungal sepsis-specific gene set; the bacterial sepsis GSVA index may be a reliable biomarker for bacterial sepsis.

## Data Availability

The data used to support the findings of this study are available from the corresponding author upon request.
